# Simple Respiratory Mask

**DOI:** 10.3201/eid1206.051468

**Published:** 2006-06

**Authors:** Virginia M. Dato, David Hostler, Michael E. Hahn

**Affiliations:** * University of Pittsburgh, Pittsburgh, Pennsylvania, USA

**Keywords:** Mask, Respiratory protection, Respiratory Protective Devices, Influenza, Avian, letter

**To the Editor:** The US Department of Labor recommends air-purifying respirators (e.g., N95, N99, or N100) as part of a comprehensive respiratory protection program for workers directly involved with avian influenza–infected birds or patients (*1*). N95 respirators have 2 advantages over simple cloth or surgical masks; they are >95% efficient at filtering 0.3-μm particles (smaller than the 5-μm size of large droplets—created during talking, coughing, and sneezing—which usually transmit influenza) and are fit tested to ensure that infectious droplets and particles do not leak around the mask (*2**–**4*). Even if N95 filtration is unnecessary for avian influenza, N95 fit offers advantages over a loose-fitting surgical mask by eliminating leakage around the mask.

The World Health Organization recommends protective equipment including masks (if they not available, a cloth to cover the mouth is recommended) for persons who must handle dead or ill chickens in regions affected by H5N1 (*5*). Quality commercial masks are not always accessible, but anecdotal evidence has showed that handmade masks of cotton gauze were protective in military barracks and in healthcare workers during the Manchurian epidemic (*6**,**7*). A simple, locally made, washable mask may be a solution if commercial masks are not available. We describe the test results of 1 handmade, reusable, cotton mask.

For material, we choose heavyweight T-shirts similar to the 2-ply battle dress uniform T-shirts used for protective masks against ricin and saxitoxin in mouse experiments (*8*). Designs and T-shirts were initially screened with a short version of a qualitative Bitrex fit test (*9*) (Allegro Industries, Garden Grove, CA, USA). The best were tested by using a standard quantitative fit test, the Portacount Plus Respirator Fit Tester with N95-Companion (TSI, Shoreview, MN, USA) (*10*). Poor results from the initial quantitative fit testing on early prototypes resulted in the addition of 4 layers of material to the simplest mask design. This mask is referred to as the prototype mask (Figure).

**Figure:**
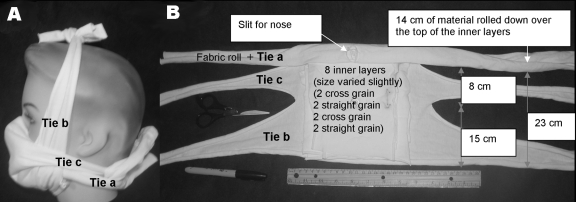
Prototype mask. A) Side view, B) Face side. This mask consisted of 1 outer layer (≈37 cm × 72 cm) rolled and cut as in panel B with 8 inner layers (<18 cm^2^) placed inside (against the face). The nose slit was first placed over the bridge of the nose, and the roll was tied below the back of the neck. The area around the nose was adjusted to eliminate any leakage. If the seal was not tight, it was adjusted by adding extra material under the roll between the cheek and nose or by pushing the rolled fabric above or below the cheekbone. Tie b was tied over the head. A cloth extension was added if tie b was too short. Finally, tie c was tied behind the head. The mask was then fit tested.

A Hanes Heavyweight 100% preshrunk cotton T-shirt (made in Honduras) (http://www.hanesprintables.com/Globals/Faq.aspx) was boiled for 10 minutes and air-dried to maximize shrinkage and sterilize the material in a manner available in developing countries. A scissor, marker, and ruler were used to cut out 1 outer layer (≈37 × 72 cm) and 8 inner layers (<18 cm^2^). The mask was assembled and fitted as shown in the Figure.

A fit factor is the number generated during quantitative fit testing by simulating workplace activities (a series of exercises, each 1 minute in duration). The Portacount Plus Respirator Fit Tester with N95-Companion used for the test is an ambient aerosol instrument that measures aerosol concentration outside and inside the prototype mask. The challenge agent used is the ambient microscopic dust and other aerosols that are present in the air.

A commercially available N95 respirator requires a fit factor of 100 to be considered adequate in the workplace. The prototype mask achieved a fit factor of 67 for 1 author with a Los Alamos National Laboratory (LANL) panel face size of 4, a common size. Although insufficient for the workplace, this mask offered substantial protection from the challenge aerosol and showed good fit with minimal leakage. The other 2 authors with LANL panel face size 10, the largest size, achieved fit factors of 13 and 17 by making the prototype mask inner layers slightly larger (22 cm^2^).

We do not advocate use of this respirator in place of a properly fitted commercial respirator. Although subjectively we did not find the work of breathing required with the prototype mask to be different from that required with a standard N95 filtering facepiece, persons with respiratory compromise of any type should not use this mask. While testers wore the mask for an hour without difficulty, we cannot comment on its utility during strenuous work or adverse environmental conditions.

We showed that a hand-fashioned mask can provide a good fit and a measurable level of protection from a challenge aerosol. Problems remain. When made by naive users, this mask may be less effective because of variations in material, assembly, facial structure, cultural practices, and handling. No easy, definitive, and affordable test can demonstrate effectiveness before each use. Wearers may find the mask uncomfortable.

We encourage innovation to improve respiratory protection options. Future studies must be conducted to determine levels of protection achieved when naive users, following instructions, produce a similar mask from identical or similar raw materials. Research is needed to determine the minimal level of protection needed when resources are not available for N95 air-purifying respirators since the pandemic threat from H5N1 and other possible influenza strains will exist for the foreseeable future.

## References

[1] Occupational Safety and Health Administration. Guidance for protecting workers against avian flu. [cited 2005 Oct 23]. Available from http://www.osha.gov/dsg/guidance/avian-flu.html

[2] National Institute for Occupational Safety and Health. 42 CFR Part 84 Respiratory protective devices. 1995 [cited 2005 Oct 23]. Available from http://www.cdc.gov/niosh/pt84abs2.html

[3] Garner JS. Guideline for isolation precautions in hospitals. The Hospital Infection Control Practices Advisory Committee. Infect Control Hosp Epidemiol. 1996;17:53–80. 10.1086/647190 8789689

[4] Centers for Disease Control and Prevention. Laboratory performance evaluation of N95 filtering facepiece respirators. MMWR Morb Mortal Wkly Rep. 1996;1998:1045–9. 9869077

[5] World Health Organization Regional Office for the Western Pacific. Advice for people living in areas affected by bird flu or avian influenza. 2004 Nov 8 [cited 2005 Oct 22]. Available from http://www.wpro.who.int/NR/rdonlyres/04FA6993-8CD1-4B72-ACB9-EB0EBD3D0CB1/0/Advice10022004rev08112004.pdf

[6] Capps JA. Measures for the prevention and control of respiratory infections in military camps. JAMA. 1918;71:448–50. 10.1001/jama.1918.26020320008010a

[7] Kool JL. Risk of person-to-person transmission of pneumonic plague. Clin Infect Dis. 2005;40:1166–72. 10.1086/428617 15791518

[8] Darling RG. Biological warfare and bioterrorism. Slides 47 and 48. [cited 2006 Mar 19]. Available from http://www.regionsem.org/~trjoing/papers/123456/clr/Slides%20with %20Notes/Biological%20Warfare%20&%20Bioterrorism.pdf

[9] Occupational Safety and Health Administration. Fit testing procedures (mandatory)–1910.134 App A. [cited 2006 Jan 21]. Available from http://www.osha.gov/pls/oshaweb/owadisp.show_document?p_table= STANDARDS&p_id=9780&p_text_version=FALSE#Appendix%20A

[10] TSI incorporated. How to quantitatively fit test filtering-face piece respirators using a TSI Portacount Plus and N95-Companion (ITI-054) c2006. [cited 2006 Jan 21]. Available from http://www.tsi.com/AppNotes/appnotes.aspx?Pid=33&lid=445&file=iti_054

